# Diagnostic Value of a Protocolized In-Depth Evaluation of Pediatric Bone Marrow Failure: A Multi-Center Prospective Cohort Study

**DOI:** 10.3389/fimmu.2022.883826

**Published:** 2022-04-27

**Authors:** Khaled Atmar, Claudia A. L. Ruivenkamp, Louise Hooimeijer, Esther A. R. Nibbeling, Corien L. Eckhardt, Elise J. Huisman, Arjan C. Lankester, Marije Bartels, Gijs W. E. Santen, Frans J. Smiers, Mirjam van der Burg, Alexander B. Mohseny

**Affiliations:** ^1^ Department of Pediatric Hematology and Stem Cell Transplantation, Willem-Alexander Children’s Hospital, Leiden University Medical Center, Leiden, Netherlands; ^2^ Department of Clinical Genetics, Leiden University Medical Center, Leiden, Netherlands; ^3^ Department of Pediatric Hematology, Beatrix Children’s Hospital, University Medical Center Groningen, Groningen, Netherlands; ^4^ Department of Pediatric Hematology, Amsterdam University Medical Center, Amsterdam, Netherlands; ^5^ Department of Pediatric Hematology, Erasmus Medical Center, Sophia Children’s Hospital, Rotterdam, Netherlands; ^6^ Department of Pediatric Hematology, Wilhelmina Children’s Hospital, University Medical Center Utrecht, Utrecht, Netherlands

**Keywords:** bone marrow failure, BMF, aplastic anemia, AA, cytopenia, next-generation sequencing, diagnostics

## Abstract

**Background:**

Severe multilineage cytopenia in childhood caused by bone marrow failure (BMF) often represents a serious condition requiring specific management. Patients are at risk for invasive infections and bleeding complications. Previous studies report low rates of identifiable causes of pediatric BMF, rendering most patients with a descriptive diagnosis such as aplastic anemia (AA).

**Methods:**

We conducted a multi-center prospective cohort study in which an extensive diagnostic approach for pediatric patients with suspected BMF was implemented. After exclusion of malignant and transient causes of BMF, patients entered thorough diagnostic evaluation including bone marrow analysis, whole exome sequencing (WES) including copy number variation (CNV) analysis and/or single nucleotide polymorphisms (SNP) array analysis. In addition, functional and immunological evaluation were performed. Here we report the outcomes of the first 50 patients (2017-2021) evaluated by this approach.

**Results:**

In 20 patients (40%) a causative diagnosis was made. In this group, 18 diagnoses were established by genetic analysis, including 14 mutations and 4 chromosomal deletions. The 2 remaining patients had short telomeres while no causative genetic defect was found. Of the remaining 30 patients (60%), 21 were diagnosed with severe aplastic anemia (SAA) based on peripheral multi-lineage cytopenia and hypoplastic bone marrow, and 9 were classified as unexplained cytopenia without bone marrow hypoplasia. In total 28 patients had undergone hematopoietic stem cell transplantation (HSCT) of which 22 patients with an unknown cause and 6 patients with an identified cause for BMF.

**Conclusion:**

We conclude that a standardized in-depth diagnostic protocol as presented here, can increase the frequency of identifiable causes within the heterogeneous group of pediatric BMF. We underline the importance of full genetic analysis complemented by functional tests of all patients as genetic causes are not limited to patients with typical (syndromal) clinical characteristics beyond cytopenia. In addition, it is of importance to apply genome wide genetic analysis, since defects in novel genes are frequently discovered in this group. Identification of a causal abnormality consequently has implications for the choice of treatment and in some cases prevention of invasive therapies.

## Introduction

Bone marrow failure (BMF) is a heterogeneous group of hematological disorders with overlap between inherited bone marrow failure syndromes (IBMFS), inborn errors of immunity (IEI), aplastic anemia (AA), and myelodysplastic syndromes (MDS) (1–6). Mutations in a growing number of genes have been described in patients with IBMFS (7–9). However, most studies have focused on patients with specific disease entities based on distinct phenotypic characteristics. These studies therefore did not provide a wider perspective of the underlying diagnoses for the total group of patients with BMF (9–12). Timely and accurate identification of an underlying cause for BMF is essential to ensure appropriate medical care (13, 14). Early diagnosis allows for risk-adapted organ monitoring, cancer risk assessment and family counseling depending on the underlying disease. Moreover, recognizing a genetic cause of BMF is the crucial step in preventing inappropriate administration of immunosuppressive therapy (IST), timely initiation of hematopoietic stem cell transplantation (HSCT), the selection of unaffected HLA-matched sibling donors, and importantly adapting the conditioning regimen to avoid toxicity arising from underlying genetic defects (7, 10, 15, 16).

In up to 50% of pediatric BMF an underlying germline causative mutation is suspected depending on the presence of other distinctive physical features in addition to hematological abnormalities (7, 17, 18). This group of IBMFS comprises hematological disorders characterized by ineffective hematopoiesis, predisposition to cancer, and often by congenital malformations (17). IBMFS may present with single cell cytopenia, e.g., Diamond-Blackfan anemia (DBA) or severe congenital neutropenia (SCN). Additionally, they may evolve towards or present with pancytopenia. The underlying mechanism of defective hematopoiesis is dependent on functional consequences of a genetic defect, such as an impaired DNA repair system in Fanconi Anemia (FA) or a telomere biology disorder (TBD), such as in Dyskeratosis Congenita (DKC) (19, 20). In addition to the classical IBMFS, indirect damage to the hematopoietic stem cells due to constitutional gene defects such as *CTLA4* or *DADA2* mutations, can also cause pancytopenia (21, 22). Importantly, IBMFS can be associated with hematological and non-hematological malignant propensity, such as in FA. This also applies to mutations in *GATA2*, *ETV6*, and *SRP72* associated with an increased risk of progression to leukemia (23–26). Unlike IBMFS, in childhood myelodysplastic syndrome (MDS) somatic chromosomal alterations and mutations are related to an increased frequency of malignant progression (27, 28).

Currently, guidelines for genetic screening in BMF are inconsistent and the extensiveness of genetic testing is mostly guided by the presence of extra-hematological physical abnormalities related to IBMFS and/or a positive family history (29). Consequently, an underlying cause for pediatric BMF patients is often lacking at presentation, with reported detection rates of 5%-50% depending on the investigated population and the type of diagnostic approach (7, 18). Thereby, most pediatric patients are categorized as severe aplastic anemia (SAA) or unexplained cytopenia without bone marrow hypoplasia at the initiation of therapy (mostly HSCT).

Aplastic anemia (AA) is a descriptive diagnosis defined as pancytopenia in combination with morphologic and histologic features of a hypocellular bone marrow (4, 18). AA terminology can be confusing as most patients present with multiple lineage cytopenia and not only anemia. Moreover, “aplastic” only represents the inability of the bone marrow to produce blood cells independent of the underlying pathophysiologic mechanisms. Thereby, AA is merely an umbrella-term for all BMF syndromes with undiscovered etiology. Disease severity is classified in three groups (moderate, severe and very severe) based on the severity of peripheral cytopenia and hypoplasia of the bone marrow according to WHO definitions (30).

With increasing application of full genomic sequencing methods, more genetic defects will be found related to the underlying cause of BMF. However, currently for most patients an identifiable (genetic) cause is not detected. BMF in this group is often hypothesized to be driven by an immunologic etiology. The role of immune dysregulation is supported by the identification of oligoclonal expanded T-cell populations in experimental settings (31, 32). Several related mechanisms have been suggested, including CD8+CD57+ oligoclonal T-cells with direct cytotoxic activity (31), secretion of different inflammatory cytokines such as interferon-γ (IFN-γ) (33), immune disarrangement by increased T-helper type 17 cells (34) or reduced regulatory T cells (Tregs) (35), and associative correlations with certain HLA types (32, 36). Another sub entity of AA is caused or associated with paroxysmal nocturnal hematuria (PNH), frequently found in adult AA patients (18, 37, 38). Recently diagnostic and prognostic value of PNH was reported in AA and MDS patients (39).

The great complexity of overlapping conditions within the entity of BMF requires a structured diagnostic approach which is currently lacking. Additionally, emphasis within current diagnostics needs to be shifted towards the potential significance of genetic evaluation in an unbiased manner. Therefore, we have developed a renewed diagnostic protocol for all pediatric patients suspected of BMF in which genetic and functional diagnostics including WES and chromosomal analyses play an integral role. To evaluate the diagnostic value of such an approach, we have conducted a prospective multi-center cohort study in which we report the results in the first 50 patients since implementation of this renewed diagnostic procedure.

## Materials and Methods

### Patients

At the Leiden University Medical Center (LUMC) Aplastic Anemia and Bone Marrow Failure Center and reference laboratory, we receive samples from most Dutch pediatric and adult patients with suspicion of BMF. The suspicion of BMF by the referring pediatric hematologist is often based on the absence of hemolysis or antibodies against blood cells, decreased reticulocytes number despite anemia, increased thrombopoietin (TPO), cytopenia present from birth, extra-hematological symptoms of IBMFS and/or positive family history.

**Figure 1:**
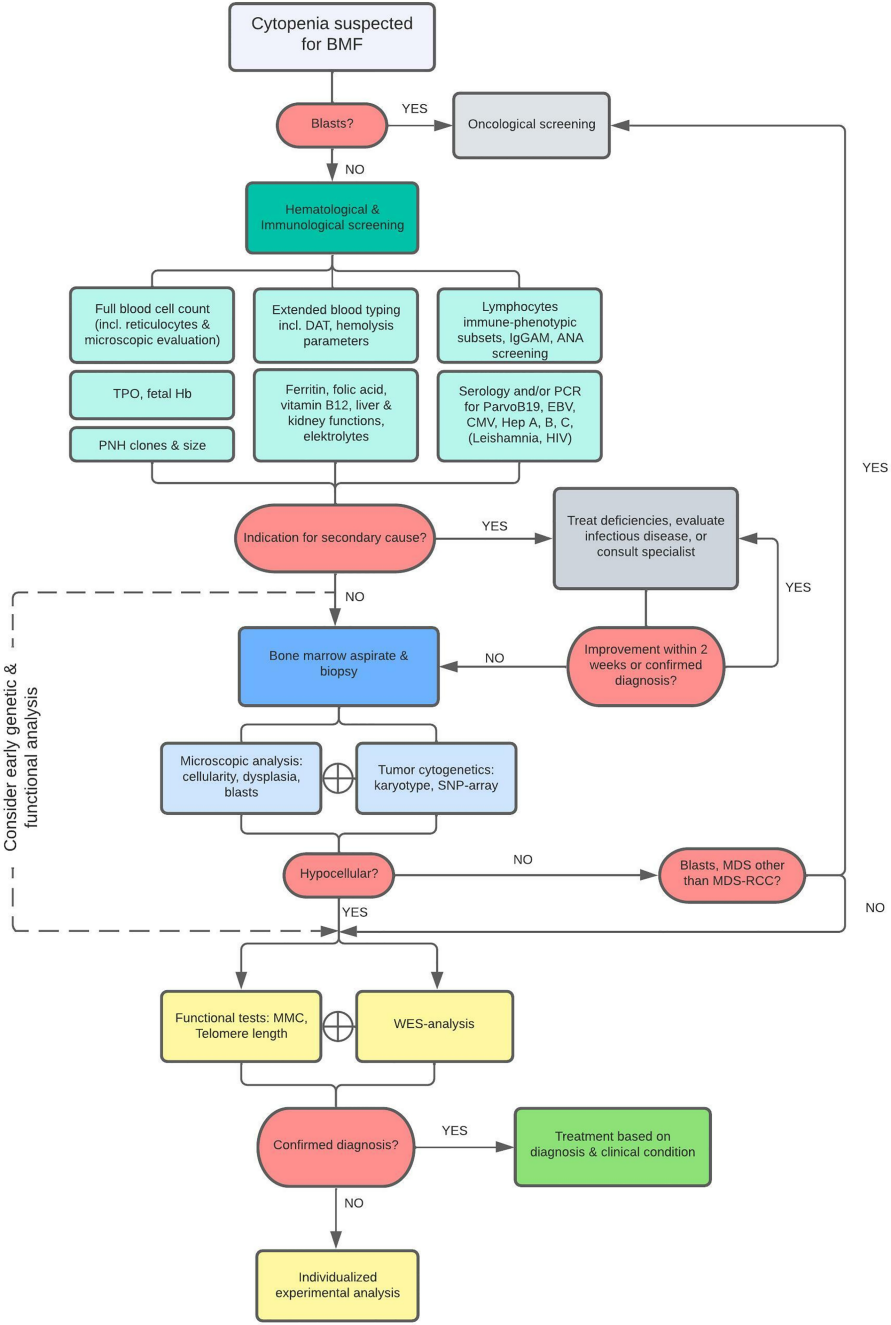
Diagnostic Flowchart. As illustrated, patients with cytopenia suspected for BMF are first screened for leukemia or other malignant disease and if indicated, patients were referred to a pediatric oncological center. Otherwise, patients were examined for specific causes related to secondary BMF. Diagnostics were followed by bone marrow analysis within 2-3 weeks when no cause for cytopenia was found or no improvement was achieved after initiation of treatment. Bone marrow aspirate and biopsy are evaluated and additionally cytogenetically assessed by karyotyping and SNP-array analysis to detect chromosomal abnormalities. Finally, a BMF filter-based WES and functional analyses are performed in all patients. The scope of WES was expanded when no mutations were found after the primary analysis if a constitutional origin of BMF was still highly suspected (e.g., IEOI-filter, Trio-WES, Open-WES).

After exclusion of malignant bone marrow disease and transient and/or reversible causes of BMF by a first step local diagnostic approach, patients were included for full evaluation by our protocol (**Figure 1**). Fifty patients from four Dutch Academic Medical Centers aged between 0-18 years with suspected BMF without an identifiable cause after first-line diagnostics were analyzed by this protocol and data was prospectively collected from 2017 and 2021. All patients had cytopenia in at least one lineage (neutrophil count < 1.5 x109/L and/or platelet count < 150x109/L and/or reticulocytopenia). Only if indicated, results of blood tests and bone marrow examination were reviewed by a national BMF expert review team including the referring hematologist, reference pathologist, cytologist, clinical and laboratory geneticist, and transplantation hematologist. In addition to blood and bone marrow characteristics, any physical abnormality possibly related to BMF was recorded (e.g., growth, skeletal, neurological, and visceral abnormalities), and/or a family history (consanguinity or family history of hematological disorder). In case of trio and unfiltered genetic analysis or tissue/data banking for patients undergoing HSCT at the Pediatric Transplantation Unit (LUMC), the procedure was always preceded by written informed consent by the patients and/or parents in accordance with the Declaration of Helsinki and Dutch law.

### Blood Tests

The following diagnostic tests were performed (**Figure 1**):

• Full blood cell count including reticulocytes count, indices, and microscopic evaluation for the presence of blasts, blood group type and screen, - ferritin, zinc protoporphyrin (ZPP) if available, folic acid, vitamin B12, liver and kidney functions, hemolysis and tumorlysis parameters (including Direct Antiglobulin Test; DAT) and electrolytes, - serology and/or PCR tests for ParvoB19, EBV, CMV, hepatitis A, B, C, Leishmania (and HIV if indicated)
• Thrombopoietin (TPO), fetal Hb, immunoglobulins, and lymphocytes immune-phenotypic subsets, Antinuclear Antibody (ANA) screening and Paroxysmal Nocturnal Hemoglobinuria (PNH) clones.

These diagnostic tests were followed by bone marrow analysis within 2-3 weeks on average if no explanatory cause for the cytopenia was detected.

### Bone Marrow

Bone marrow aspirates and biopsies were evaluated by a local experienced cytologist and pathologist and re-evaluated by the central referral cytologist and pathologist if needed. This evaluation included analysis of several histopathological criteria in the bone marrow: 1) cellularity (percentage, adjusted for age); 2) maturation of cells in all lineages; 3) percentage of blasts (if present); and 4) signs of dysplasia, including those specific for refractory cytopenia of childhood (ROC) (40, 41). Cytogenetic analysis of the bone marrow aspirates included at least karyotyping and evaluation of the myelodysplastic syndrome associated chromosomal aberrations including monosomy 5 or 7 and trisomy 8. However, to detect other chromosomal abnormalities beyond these, SNP array analysis (CytoScan^®^ HD Array, Affymetrix) was performed for most patients for the detection of novel or smaller chromosomal aberrations. In addition, mutation analysis of bone marrow tissue was considered to investigate the presence of somatic mutations.

### Whole Exome Sequencing

WES was applied for all patients for the detection of BMF related variants. All sample analyses were performed by two laboratory specialists (CR and EN) at the LUMC. Exomes were captured using the Agilent SureSelect Human All Exon V7 capture library kit (Agilent, Santa Clara, USA) accompanied by Illumina paired end Sequencing on the NovaSeq 6000 System (Illumina, San Diego, USA). An in-house sequence analysis pipeline (Modular GATK-Based Variant Calling Pipeline, MAGPIE) based on read alignment using Burrows-Wheeler Alignment (BWA-MEM) and variant calling using the Genome Analysis Toolkit (GATK) was used to align reads and call variants on the generated BAM files. Variants were subsequently annotated using the Variant Effect Predictor. After annotation, variants with an allele frequency of > 5% in the Genome Aggregation Database (gnomAD) were excluded from further interpretation. The ACMG classification system is used for the interpretation and classification of sequence variants.

To limit the number of variants of unknown significance (VUS), primarily only known genes were analyzed by applying a defined gene filter to the WES data containing 172 genes (BMF gene panel, **Supplementary 1**) and HPO terms based on the type of cytopenia and any other clinical symptoms (Moon software, Diploid). This filter also includes genes associated with predisposition to malignant disease such as those caused by mutations in *GATA2, ETV6* and *SAMD9/SAMD9L* (10). In addition, genes involved in overlap syndromes presenting by both immune deficiency or dysregulation and cytopenia such as CTLA-4 haploinsufficiency were included. Moreover, the applied filter was expanded by adding genes related to pediatric immune deficiencies (PID) when no pathogenic variants were found after the primary analysis while immune deficiency was suspected. In addition, copy number variant (CNV) analysis of the WES data was performed for the detection of deletions and/or duplications using NxClinical (BioDiscovery).

This approach allowed for reanalysis of the data when the first analysis revealed no mutations while the clinical suspicion for an underlying congenital mutation was high. When in a patient with high suspicion of IBMFS based on clinical findings (other than cytopenia) no genetic alterations were found by this approach and concordant functional analyses were normal, the WES data was analyzed beyond the gene filter (“open-WES”) and in comparison, with parental or sibling data (“Trio-WES”), after counseling by a clinical geneticist.

### Functional Analyses

Genomic analysis was complemented by functional testing for DNA breakage defects (screening for FA) by evaluating chromosomes breakage after mitomycin C exposure (Laboratory for Medical Immunology, AMC, the Netherlands) and telomere length measurement by Flow FISH (42) (Repeat Diagnostics, Aachen, Germany) in centralized laboratories.

### Statistical Analysis

Statistical analysis was executed by using IBM SPSS version 25.0. The Mann-Whitney U test was performed for comparison of non-parametrical data. A value of p < 0.05 was considered statistically significant.

## Results

### Blood and Bone Marrow Examination

The clinical characteristics of the cohort included in this study are presented in **Table 1**. Both genders were equally represented. The median age at the start of the diagnostic evaluation was eight years (range one month – eighteen years). Within this cohort twenty-eight patients (56%) presented with pancytopenia. Eight patients (16%) had cytopenia in two hematopoietic lineages of which the combination anemia with neutropenia (N=3), anemia with thrombocytopenia (N=3), and neutropenia with thrombocytopenia (N=2) were distributed nearly equally. Furthermore, 12/50 (24%) patients presented with non-hematological clinical manifestations in addition to cytopenia of which facial dysmorphias and growth/developmental disorders occurred most frequently in seven and five patients, respectively. First-line peripheral blood tests after exclusion of malignant disorders revealed no causative defect for the cytopenia (**Figure 1**). Other known causes of secondary BMF such as (viral) infections or malnutrition were excluded in all patients. In three patients, cytopenia was preceded by acute liver disease of unknown origin, a condition described as hepatitis associated aplastic anemia (HAAA) (43). PNH clones were only found in four patients of which three 0,1-1% and one 8%. Within this cohort, 27 patients had hypoplastic bone marrow (**Figure 1**). Microscopic analysis of the bone marrow showed signs of dysplasia in two patients. One was related to a *SAMD9* mutation. In this patient the percentage of dysplasia was increasing at subsequent bone marrow analyses and lead to the decision for HSCT to prevent MDS/leukemia evolution. In the other patient, dysplasia was recurrently limited to <10% and explained by the 11q deletion, a known phenomenon of Jacobsen syndrome. Despite thorough bone marrow aspiration and biopsy analysis, according to previous published histopathologic criteria (41) no cases of refractory cytopenia of the childhood (MDS-RCC) were identified.

**Table 1:** Patient characteristics.

Characteristics	Value
**Total patients, N, (%)**	50
** Male**	- 29 (58)
** Female**	- 21 (42)
**Age, median (range)**	8 years (1 month – 18 years)
**Age in years, N, (%)**	
** ≤ 2**	8 (16)
** 3-11**	28 (56)
** ≥ 12**	14 (28)
**Nr. of cytopenias, N, (%)**	
** Pancytopenia**	28 (56)
** Cytopenia, 1 lineage**	14 (28)
** Anemia**	- 3
** Neutropenia (< 1.5 x10^9^/L)**	- 1
** Thrombocytopenia (< 150x10^9^/L)**	- 10
** Cytopenia, 2 lineages**	8 (16)
** Anemia + Neutropenia**	- 3
** Anemia + Thrombocytopenia**	- 3
** Neutropenia + Thrombocytopenia**	- 2
**Physical abnormalities, N, (%)**	
** Facial dysmorphias**	7 (14)
** Growth restriction**	4 (8)
** Cardiac disorder**	2 (4)
** Skin, nail, or hair abnormalities**	2 (4)
** Skeletal malformation (e.g., short thumbs)**	2 (4)
** Splenomegaly**	1 (2)
** (Neuro-)muscular disorder**	1 (2)
** Developmental disorder**	1 (2)

### Immunological Screening

**Figure 2:**
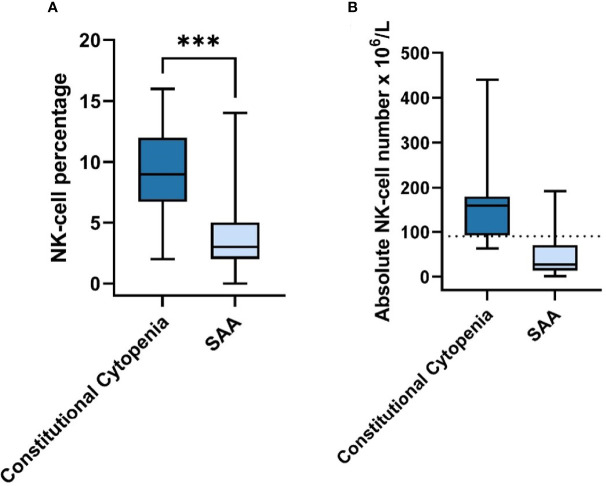
NK-cell Percentage & Absolute Number in Constitutional Cytopenia vs. SAA. **(A)** NK-cell percentage relative to total lymphocyte count in peripheral blood of patients with identified causal abnormality (N=14) compared to SAA-patients (N=21) is shown. Data is represented by a boxplot displaying the median, IQR, minimum and maximum. Statistical significance was determined using the Mann-Whitney U test. ***P < 0.001. **(B)** Absolute NK-cell number x 10^6^/L in peripheral blood of patients with identified causal abnormality (N=14) compared to SAA-patients (N=21). Median age for Constitutional Cytopenia vs. SAA groups was 7.0 years (IQR=7,95) vs. 10.3 (IQR=9.0). Data is represented by a boxplot displaying the median, IQR, minimum and maximum, and lower limit of reference range for the age of 5-10 years (dotted line).

To assess characteristics of autoimmunity in our patients, the protocol included ANA screening and extensive lymphocytes’ immune-phenotypic subsets (**Figure 1**). In none of the patients auto-antibodies were found and all T- and B-cell subsets were present in normal numbers according to age adjusted normal ranges (data not shown). However, NK cell numbers were significantly lower in patients without an identifiable cause for BMF, especially in the SAA group (**Figure 2**). The individual subsets were quantitatively compared between patients with an identified cause for BMF (N=14/20) and SAA (N=21) group. The median percentage of NK-cells in peripheral blood lymphocytes was significantly lower in the SAA group compared to the constitutional cytopenia group; 9.0 (IQR = 5.25) vs. 3.0 (IQR = 3) (P < 0.05).

### (Cyto)Genetic, and Functional Analysis

In 20/50 (40%) patients a (likely) causative diagnosis was established. WES analysis of all patients revealed a (likely) pathogenic variant in fourteen patients (28%) (**Table 2**). This group will be referred to as constitutional cytopenias. In this group 21 genetic changes were found, most of which were substitutions (N=16), in addition to four deletions and one duplication. In addition to mutations, chromosomal deletions were identified by SNP array analysis in four cases (8%) (**Table 3**). These were localized twice on chromosome 11, and in two patients on chromosomes 12 and 15. The number of protein coding genes at these regions are 6 in the smallest deletion to 98 in the largest. Even though no genetic abnormality had been found in two patients, functional analysis showed significantly shorter telomeres in these patients in addition to other phenotypic characteristics consistent with a TBD such as distinctive facial features, short thumbs, nail abnormalities and small stature. One patient with a known mutation in the *FANCC* gene also had a concordantly abnormal MMC test.

**Table 2:** List of genes and identified mutations.

Pt	Gene	OMIM nr.	Zygosity	CHR	Change in DNA	Change in protein	Detection modality	Parental occurrence	Class	Biological profile	Functional profile	Diseases associated
1	***ABCG8*** NM_022437.2	605460	Compound heterozygous	Chr2	c.1083G**>**A c.1715T**>**C	p.Trp361* p.Leu572Pro	HPO-terms	No	P LP	ATP-bindingcassette (ABC) transporter	Sterol homeostasis	Sitosterolemia, Gallbladder disease
2			homozygous	Chr2	c.1715T**>**C	p.Leu572Pro	HPO-terms	-	LP			
3	***ACTB*** NM_001101.3	102630	Heterozygous	Chr7	c.1084dup	p.Tyr362Leu fs*2	BMF-filter	-	LP	Cytoplasmic actin	cell motility, contraction, gene transcription, DNA-repair, platelet activation/aggregation	Dystonia, Juvenile-Onset, Baraitser-Winter Syndrome 1
4	***FANCC*** NM_000136.2	613899	Homozygous	Chr9	c.67del	p.Asp23Ile fs*23	BMF-filter	-	P	DNA-repair protein	Interstrand DNA cross-link repair	Fanconi Anemia
5	***GNE*** NM_001128227.2	603824	Compound heterozygous	Chr9	c.173C**>**T c.491T**>**C	p.Pro58Leu p.Ile146Thr	Trio-WES	Yes	P LP	Bifunctional UDP-N-acetylglucosamine 2-epimerase/N-acetylmannosamine kinase	Regulates and initiates biosynthesis of N-acetylneuraminic acid (required for normal sialylation in hematopoietic cells)	Nonaka Myopathy, Sialuria
6	***HBB*** NM_000518.4	141900	Heterozygous, *de novo*	Chr11	c.425T**>**C	p.Leu142Pro	HPO-terms	-	P	Hemoglobin subunit	Oxygen transport, involved in megakaryocyte development and platelet production	Beta-Thalassemia, Sickle Cell Anemia.
7	***MPL*** NM_005373.2	159530	Compound heterozygous	Chr1	c.378del c.1000T**>**C	p.Phe126Leufs*5 p.Cys334Arg	BMF-filter	-	P LP	Thrombopoietin receptor	Regulation of megakaryopoiesis and platelet production	Myelofibrosis, Congenital amegakaryocytic thrombocytopenia
8			Homozygous	Chr1	c.23T**>**G	p.Met8Arg	BMF-filter	-	LP			
9	***RPL35A*** NM_000996.2	180468	Heterozygous	Chr3	c.125A**>**G	p.Tyr42Cys	BMF-filter	-	P	Ribosomal protein	Proliferation and viability of hematopoietic cells	Diamond-Blackfan Anemia
10			Heterozygous	Chr3	c.82_84del	p.Leu28del	BMF-filter	-	P			
11	***SAMD9*** NM_017654.3	610456	Heterozygous, *de novo*	Chr7	c.773A**>**C	p.Glu258Ala	BMF-filter	No	LP	Sterile alpha motif domain-containing protein	Regulator of cell proliferation, apoptosis, inflammatory signaling, endosome fusion	Mirage Syndrome, Familial tumoral calcinosis, Monosomy 7 myelodysplasia and leukemia syndrome
12	***SRC*** NM_005417.4	190090	Heterozygous, *de novo*	Chr20	c.1579G**>**A	p.Glu527Lys	HPO-terms	No	P	Proto-oncogene tyrosine-protein kinase	Participates in embryonic development, cell growth, gene transcription, immune response, cell adhesion, cell cycle progression, apoptosis, migration, platelet activation/aggregation	Thrombocytopenia, Colorectal cancer
13	***TERT*** NM_198253.2	187270	Compound heterozygous	Chr5	c.2005C**>**T c.3208G**>**A	p.Arg669Trp p.Val1070Met	BMF-filter	Yes	P VUS	Telomerase reverse transcriptase	Chromosomal replication	Dyskeratosis Congenita, Pulmonary fibrosis, Acute Myeloid Leukemia, Cutaneous Melanoma
14			Heterozygous, *de novo*	Chr5	c.1796G**>**A	p.Arg599Gln	BMF-filter	No	LP			

CHR, chromosome; P, pathogenic; LP, likely pathogenic; VUS, variant of unknown significance.

**Table 3:** List of chromosomal aberrations.

Pt	CHR	Change in DNA	Number of protein coding genes affected	Detection modality	Class	Diseased associated
**15**	Chr11	Germline deletion from BP 126.908.896 – 134.938.470	32	SNP-array	P	Jacobsen syndrome
**16**	Chr11	Deletion from BP 123.307.968 – 134.938.470	98	SNP-array	P	Jacobsen syndrome
**17**	Chr12	Germline deletion from BP 56.354.128 – 56.586.298 bp	13	SNP-array	P	Diamond-Blackfan Anemia
**18**	Chr15q3.3	Deletion from BP 31.112.920 – 32.444.044	6	SNP-array	LP	15q13.3 microdeletion syndrome

CHR, chromosome; P, pathogenic; LP, likely pathogenic; BP, base pair.

**Figure 3:**
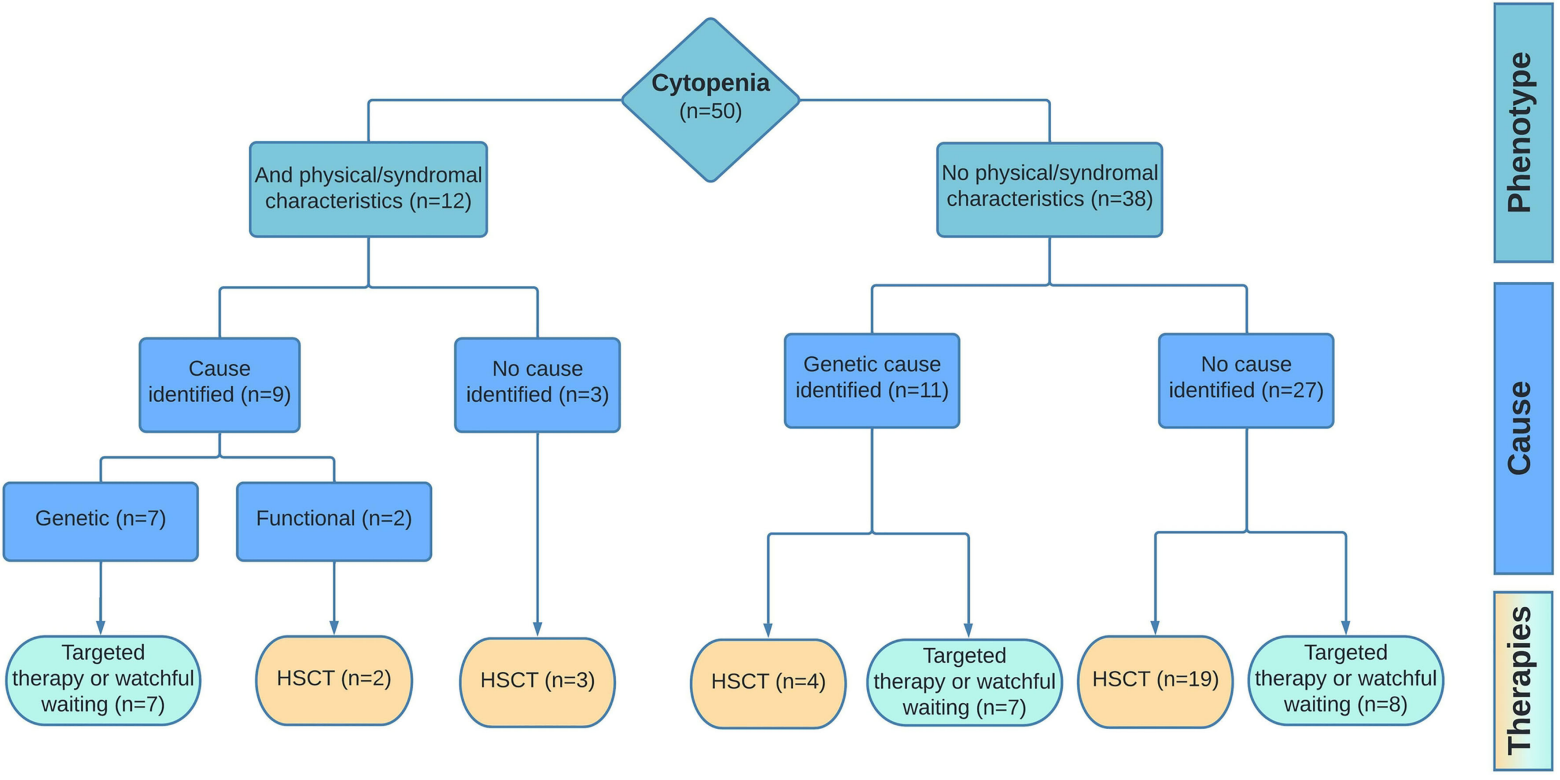
Diagnostic outcome and management. Results of the diagnostic pipeline and the implemented therapies are shown for patients with cytopenia and additional (phenotypic/syndromal) abnormalities vs patient with isolated cytopenia. Targeted therapy includes treatment options as corticosteroids, androgens, or dietary interventions.

No causative diagnosis was made in the remaining thirty patients. In this group, 21 patients met the criteria for SAA and the remaining nine patients had unexplained cytopenia in the absence of bone marrow hypoplasia. To explore the detection rate of in-depth genetic diagnostics in all patients rather than only those with phenotypic/syndromal abnormalities, we evaluated the number of cases in which a genetic mutation was found in the groups with (N=12) and without (N=38) non-hematological clinical manifestations. In 9/12 patients (75%) with other abnormalities, a molecular/functional abnormality was found. In patients without physical abnormalities, this was the case in 11/38 patients (29%) (**Figure 3**).

### Genetic Analysis

**Table 2** displays the fourteen (likely) pathogenic variants detected by WES. In total, ten different genes were found to be involved in this group. Recurrent pathogenic variants were found in the *ABCG8*, *RPL35A*, *MPL*, and *TERT* gene. Other genes including *ACTB*, *FANCC*, *GNE*, *HBB*, *SAMD9*, and *SRC* were affected in a single patient. Of these fourteen patients, seven (50%) had a mono-allelic variant and the other half had biallelic variants based on compound heterozygosity (N=4) and homozygosity (N=3). Within the group of patients with a pathogenic variant, 9/14 (64%) were identified based on the BMF gene filter applied to the WES data. This included variants in *FANC*, *RPL35A*, *TERT*, *ACTB*, *MPL*, and *SAMD9*. In four patients, a pathogenic variant in the *SRC*, *HBB* and *ABCG8* gene was identified using HPO terms. Moreover, in one patient for whom a trio WES was performed because of syndromal features and absence of a causal genetic defect after primary analysis, a pathogenic variant was found in the *GNE* gene. In the group of patients where no causal abnormality was found, a PID filter was applied to WES data of four patients because of suspected immunodeficiency, for three patients a trio WES was performed and in one patient an open WES, however no mutations were detected.

In addition to of the (likely) pathogenic mutations, single variants of unknown significance (VUS) were found in 9/50 (18%) patients of which 6/9 in patient with another identified likely causative defect. Moreover, in two male patients, a BRCA1 mutation was identified as an incidental finding.

Pathogenic variants were found in genes encoding for proteins with a variety of properties, some of which were directly affecting viability and functioning of hematopoietic stem cells (HSC) or their differentiated hematopoietic descendants. As such, *FANCC*, *RPL35A* and *TERT* are known genes whose mutations lead to FA, DBA, and DKC, respectively known as the classical IBMFS. In 9/14 patients (64%) (likely) causative mutations were detected which are rarely or not previously reported in IBMFS, including *GNE* (N=1), *ABCG8* (N=2), *ACTB* (N=1), *HBB* (N=1), *SAMD9* (N=1), and *SRC* (N=1). A gene that is not classically associated with an IBMFS, but in which mutations are known to cause symptoms of thrombocytopenia, is *MPL* (N=2). *MPL* encodes for the thrombopoietin receptor, required for regulation of megakaryopoiesis and platelet production (43). Evidently, both patients with a mutation in this gene presented with isolated thrombocytopenia (CAMT).

*GNE* is known to play an important role in sialylation in hematopoietic cells (44). A process involving covalent addition of sialic acid to the terminal end of glycoproteins crucial for biological processes such as cell adhesion and signal transduction. To date, new *GNE* variants associated with severe thrombocytopenia are found (45). Mutations in this gene have also been associated with the rare autosomal recessive *GNE*-myopathy (46). The patient from our cohort with this mutation also presented with myopathy in addition to the combination of thrombopenia and leukopenia.

Mutations in the *ABCG8* gene have been reported to be associated with the rare autosomal recessive lipid metabolic disorder Sitosterolemia, recently linked to four cases of hemolytic anemia with signs such as stomatocytic hemolysis, macrothrombocytopenia, and splenomegaly (47). Within our cohort, the two patients with pathogenic variants in this gene presented with a combined anemia and thrombocytopenia in one patient, and pancytopenia in the other. No other physical abnormalities were identified. Variants in ACTB, which encodes for β-cytoplasmic actin, have been reported in various clinical entities. A disease primarily associated with mutations in exon 2-4 of this gene is Baraitser-Winter Cerebrofrontofacial syndrome (BWCFF) characterized by distinct facial features and developmental disorders (48). Recently, pathogenic variants in exons 5 and 6 of ACTB have been reported in six patients presenting with clinical symptoms different from BWCFF, particularly thrombocytopenia which is caused by inhibited platelet maturation as a result of a compromised microtubule organization (49). One of our patients presented with isolated thrombocytopenia alongside a patent ductus arteriosus and hitherto no signs of a developmental disorder while the other had pancytopenia in the absence of extra-hematological symptoms.

Germline mutations in the *SAMD9* and *SAMD9L* gene, located on chromosome 7, are associated with a clinical spectrum of disorders including the MIRAGE syndrome, ataxia–pancytopenia syndrome and monosomy 7 myelodysplastic syndrome. Germline gain-of-function mutations cause pancytopenia. As an escape mechanism, somatic reversions have been described, including loss of the chromosome 7 and sometimes followed by uniparental disomy of the healthy chromosome 7. However, the (intercurrent) monosomy 7 can give rise to the clonal expansion of cells with reduced or no antiproliferative effect of *SAMD9 or SAMD9*L leading to monosomy 7 and risk of malignant transformation (50–53). However, the diagnostic distinction between “protective” loss of mutated chromosome 7 to revert cytopenia and monosomy 7 with risk of clonal progression is very limited at this moment. Accordingly, our patient presented with intermittent severe cytopenia which was accompanied by monosomy 7 and uniparental disomy for which initially a watchful waiting management was appointed. However, due to progressive myelodysplasia accompanied by an increasing clone size harboring monosomy 7, after two years the treating physician and parents decided towards HSCT before the onset of malignant transformation.

A novel *HBB* mutation was identified in one patient. Previous standard hemoglobinopathy diagnostics had revealed no explanation for the anemia for which the patient was transfusion dependent more than two years prior to analysis by this protocol. WES identified a hemoglobin variant, not previously described, and now annotated as “HbGroningen”. Parents we confirmed non carries of this *de novo* mutation which causes instable hemoglobin.

Lastly, the *SRC* gene encodes for a tyrosine-protein kinase with its active form also present in platelets. A recent publication including nine patients with a gain-of-function mutation in the *SRC* gene demonstrated clinical phenotypes with signs such as thrombocytopenia, myelofibrosis, bleeding, and bone pathology (54). Our patient presented with thrombocytopenia, anemia, and splenomegaly of which the latter is commonly a result of myelofibrotic progression.

### Treatment Strategy

Among the 50 patients in this cohort 28 (56%) were treated by HSCT of which six were diagnosed with constitutional cytopenia, one patient with an unexplained, transfusion dependent cytopenia, and all 21 patients with SAA (**Figure 3**). For the remaining group 10% received supportive or tailored care in the form of corticosteroids (N=2), androgen therapy (N=1), or dietary changes to reduce sterol intake (N=2). The latter was specifically the case for both patients with the *ABCG8* mutation. One patient with unexplained cytopenia received occasional transfusions. In sixteen patients (34%), nine with constitutional cytopenia, no treatment was necessary based on their clinical condition and/or diagnosis. These patients were closely monitored in which the frequency of follow-up varied according to diagnosis and expected risks.

## Discussion

Pediatric patients with severe BMF frequently remain without a classifying diagnosis after initial etiological workup and enter treatment and follow-up programs for SAA. In this study, using comprehensive clinical, functional, and genetic analysis including WES, we provide a molecular/functional diagnosis for 40% of a pediatric BMF cohort with major implications for their clinical management. Due to the lack of a previous national consensus on the diagnostics of pediatric BMF we were unable to compare the diagnostic yield of the presented approach here to previous protocols (55, 56), in which especially the type and extend of genetic analysis were variable. In addition, not all Dutch pediatric centers evaluated pediatric patients suspected for BMF according to this protocol and not all patients were included when based on extra-hematological symptoms or family history an IBMFS was highly suspected and targeted diagnostics were performed specifically for the suspected syndrome. Hence to evaluate the diagnostic value of this protocol, we compared findings to published data on comparable groups.

In patients with hematological cytopenia without any non-hematological abnormality indicative for an underlying IBMFS or a constitutional genetic defect, a diagnosis was identified in 29% (previous reports 5-10%) (17, 18). In children suspected for BMF with additional non-hematological abnormalities, predominantly facial dysmorphic features, developmental delay, and nail/tongue abnormalities, a causative diagnosis was found in 75% (previous reports max 50%) (7, 18). Still, it remains challenging to compare findings regarding detection values between different studies, mainly due to different designs and periods of studies. Nevertheless, the importance of a comprehensive and unbiased diagnostic approach to increase the detection rate in pediatric patients with suspected BMF is evident.

Prior to the genetic and functional tests, routine peripheral blood tests take place of which the immunological screening is an important part. In our study, we observed that patients with SAA had significantly lower NK cell numbers compared to patients where a causal abnormality was identified, while other lymphocyte subsets had a similar distribution between the two groups. Low NK cell numbers in AA patients have been reported previously (57, 58). However, the etiological role of this phenomenon related to AA remains elusive. One previous study suggested skewed distribution of NK cells between BM and peripheral circulation in favor of BM as a possible explanation for the lower peripheral NK cell numbers (59).

Applying a stepwise inclusive but swift application of genome-wide genetic analysis, starting with a BMF gene panel analysis of WES data including HPO-terms, followed by Trio-WES and open exome analysis if indicated, we were able to extend the range of the common genes and mutations generally included in predefined IBMFS gene panels. Indeed, genes beyond conventional IBMFS or related to prototypical or syndromal phenotypes were identified. For example, we found mutations in *ABCG8*, *HBB*, *SRC*, *ACTB*, and *GNE* gene. Furthermore, we detected novel mutations in known IBMFS genes. This included two *TERT* mutations in patients in whom previous conventional genetic diagnostic panels did not identify a cause. Moreover, in 4 patients we found (large) chromosomal deletions identifying the cause of BMF providing crucial information for treatment choice. Especially in patients with a chromosome 11(q) deletion classified as Jacobsen syndrome, diagnosis was crucial to decide against HSCT despite clonal changes in bone marrow. In another patient with a chromosome 12 deletion, establishment of the diagnosis DBA resulted in the initiation of steroids. Interestingly, the latter genetic defect leading to DBA has not been previously described. Identification of a causal genetic defect also had broad implications for several other patients. For example, it resulted in preventing more invasive treatment options in both patients with *ABCG8* mutation since dietary changes are usually very effective (60). Moreover, as a consequence of a high-speed pipeline for the analysis of the WES data identifying an equivocal cause for the cytopenia within two weeks, invasive diagnostics such as bone marrow biopsy could be cancelled in these two patients. In some cases, as in the patient with a SAMD9 mutation and (progressive) dysplasia, it has also accelerated the route to HSCT, to prevent progression to MDS or leukemia.

Nevertheless, we were unable to identify an underlying cause for the BMF in 60% of the total cohort. Possible explanations for this include limitations of genetic analysis (related to coverage and noncoding regions) as well as the possibility of an epigenetic cause. Single cell DNA/RNA analyses can provide valuable additional diagnostic accuracy for future cohort studies to increase diagnosis detection rate.

A calculated risk of our approach was the possibility of detecting high numbers of VUS or the detection of incidental findings with clinical consequences for patients and their families. These risks were explained to all patients and parents and signed informed consent was required for unfiltered or trio genetic analysis. Although reporting VUS is highly dependent on the protocols within the genetic laboratory involved, here we show that genetic analysis by a BMF experienced laboratory and geneticists is helpful to limit VUS related difficulties. In this cohort we had a limited number of identified VUS (N=9), and these were mainly found in patients in whom a BMF causing genetic defect was already found. Therefore, after consideration the identified VUS was regarded not of importance for the clinical situation. Also, the number of incidental findings was very limited. In two male patients *BRCA1* mutations were found, and families were advised towards proper analysis and follow-up programs for family members at risk for cancer. Undoubtedly this has led to emotional stress of the parents and family members, however to our knowledge there were no other financial or other societal consequences due to these findings.

Another prior criticism to our approach was the possibility of abundance due to overlap between functional and molecular tests. However, our findings show that performed diagnostics are essential despite overlap to prevent missing diagnoses. In two patients with clear signs of telomere biology disorders (TBD) with evident short telomeres no molecular defect was found. Additionally, absence of a causal mutation, but clear functional abnormalities stimulates for more extensive genetic analysis, thus preventing a missed diagnosis. Conversely, in case a genetic defect is identified, functional testing could serve as clinical confirmation. A similar principle applies to potential overlap between WES and chromosomal analysis. In four patients without mutation after first analysis of the WES data, (larger) chromosomal deletions were found causative for BMF. Taken together, the list of diagnostics applied to this cohort should be considered the prerequisite at any center of expertise for the diagnostics and treatment of pediatric BMF.

By this prospective cohort analysis, we report a valid approach to pediatric BMF with a higher yield of diagnosis in both patients with only cytopenia as well as patients with cytopenia and phenotypic abnormalities. Our aim for future study is to unravel the causative cellular mechanism or molecular defect underlying BMF in the remaining 60% of patients. We hypothesize that also in this group variable causes underly BMF, for which our future approach will require multimodal techniques. Research at a single-cell level involving various immune cells and bone marrow hematopoietic and stromal cells could provide novel insights to improve the diagnostic detection rate in patients with suspected BMF.

## Conclusion

Our study underlines the need for a more comprehensive diagnostic approach in patients with suspected BMF, more so in an era of increasing opportunities in the field of whole exome (or genome) sequencing and molecular medicine. Timely diagnostics using this approach seemed essential to improve patient management, including decisions for invasive procedures such as HSCT. In addition to the timing and used methods for diagnostics, close collaboration between treating physicians, bone marrow specialized pathologists, geneticists, and immunologists in centers of expertise is crucial to improve diagnosis and management of BMF. Altogether, comprehensive genetic screening offers significant opportunities for personalized therapy and follow-up.

## Data Availability Statement

The datasets presented in this article are not readily available because these assays are now performed as standard clinical care based on a national protocol. Therefore, the informed consent primarily only allows for the use of data in a filtered manner. All genetic data were primarily analysed by predefined filters, as we state in material and methods section, so publishing the full raw data in public database would be against the privacy rules. Requests to access the datasets should be directed to the corresponding author.

## Ethics Statement

Ethical review and approval was not required for the study on human participants in accordance with the local legislation and institutional requirements. Written informed consent from the participants’ legal guardian/next of kin was not required to participate in this study in accordance with the national legislation and the institutional requirements.

## Author Contributions

KA, FS, AL, MB, MVB and AM drafted the aims and concept of this study and drafted the manuscript. CR, EN and GS performed and analysed genetic evaluations. LH, CE, EH, FS and AM included patients and actively participated in the expert review meetings to discuss patient findings and outcomes. All authors reviewed the manuscript and provided critical feedback. All authors read and approved the final version of manuscript.

## Conflict of Interest

The authors declare that the research was conducted in the absence of any commercial or financial relationships that could be construed as a potential conflict of interest.

## Publisher’s Note

All claims expressed in this article are solely those of the authors and do not necessarily represent those of their affiliated organizations, or those of the publisher, the editors and the reviewers. Any product that may be evaluated in this article, or claim that may be made by its manufacturer, is not guaranteed or endorsed by the publisher.
