# Supplementary material for: Diagnostic Value of a Protocolized In-Depth Evaluation of Pediatric Bone Marrow Failure: A Multi-Center Prospective Cohort Study
Source: Front Immunol. 2022 Apr 27;13:883826. doi: 10.3389/fimmu.2022.883826 (PMC9094492; doi:10.3389/fimmu.2022.883826)
Supplement: Supplementary file 1 [file DataSheet_1.pdf]

Exome
Gene panel Bone Marrow Failure (172 genes)
version 2, 09-12-2019

Leids Universitair Medisch Centrum

HGNC approved gene symbol	transcript	median depth	% covered >10x	% covered >20x	% covered >30x	% covered >50x
ABCB6	NM_005689.4	111.00	100.00	99.99	99.59	95.88
ABCB7	NM_001271696.3	91.00	100.00	99.92	99.59	97.93
ABL1	NM_005157.6	102.00	99.77	98.16	97.49	96.43
ACTB	NM_001101.5	154.00	100.00	100.00	99.80	99.50
ACTN1	NM_001130004.1	81.00	99.86	99.11	97.26	90.24
ALAS2	NM_000032.5	71.00	99.96	98.57	93.16	76.45
ANKRD26	NM_014915.2	104.00	97.43	93.90	89.45	81.40
AP3B1	NM_003664.4	99.00	98.98	97.16	95.65	90.58
ASXL1	NM_015338.6	167.00	98.78	98.71	98.42	96.97
ATRX	NM_000489.5	99.00	100.00	99.76	98.36	91.13
BCOR	NM_001123385.2	98.00	99.97	99.70	98.65	94.11
BCORL1	NM_001184772.2	76.00	99.81	98.14	92.41	81.99
BRAF	NM_004333.6	105.00	99.99	99.70	99.25	95.30
BRCA1	NM_007294.4	275.00	100.00	100.00	100.00	99.90
BRCA2	NM_000059.3	191.00	100.00	100.00	99.99	99.76
BRIP1	NM_032043.3	135.00	100.00	100.00	100.00	99.86
C15orf41	NM_001321759.2	110.00	100.00	100.00	100.00	99.52
CALR	NM_004343.3	91.00	100.00	98.41	94.67	87.37
CBL	NM_005188.4	121.00	100.00	100.00	99.98	99.80
CBLB	NM_170662.5	117.00	99.78	99.27	98.32	95.48
CDAN1	NM_138477.4	77.00	100.00	99.11	94.46	80.10
CDC42	NM_001791.4	239.00	100.00	100.00	100.00	100.00
CDKN2A	NM_000077.4	170.00	100.00	99.50	97.48	92.36
CEBPA	NM_004364.4	75.00	88.28	69.96	63.82	61.51
CECR1/ADA2	NM_001282225	125.00	100.00	100.00	99.99	99.93
CSF3R	NM_156039.3	75.00	99.67	97.21	93.58	85.38
CTC1	NM_025099.6	107.00	100.00	99.95	98.70	93.26
CTLA4	NM_005214.5	133.00	100.00	100.00	100.00	98.97
CUX1	NM_181552.4	82.00	98.16	95.51	91.62	85.53
CXCR4	NM_003467.3	172.00	100.00	100.00	100.00	99.21
CYCS	NM_018947.6	246.00	100.00	100.00	100.00	100.00
DKC1	NM_001363.5	76.00	99.70	97.99	94.81	86.99
DNAJC21	NM_001012339.3	80.00	99.99	99.57	97.95	88.57
DNMT3A	NM_022552.4	82.00	99.86	97.74	91.61	81.41
DTNBP1	NM_032122.4	88.00	100.00	99.97	99.65	92.80
ELANE	NM_001972.4	73.00	100.00	100.00	99.83	93.64
ENG	NM_001114753.2	70.00	99.84	98.42	94.65	83.14
EPG5	NM_020964.3	109.00	100.00	99.81	98.80	95.03
EPO	NM_000799.4	80.00	100.00	99.17	90.78	72.64
ERCC4	NM_005236.3	123.00	100.00	99.99	99.47	94.70
ERCC6L2	NM_020207.5	128.00	100.00	99.99	99.90	98.74
ETV6	NM_001987.5	113.00	100.00	100.00	99.94	98.69
EZH2	NM_004456.5	100.00	100.00	99.95	99.50	98.61
FANCA	NM_000135.4	88.00	100.00	99.40	96.04	82.95

HGNC approved gene symbol	transcript	median depth	% covered >10x	% covered >20x	% covered >30x	% covered >50x
FANCB	NM_001018113.3	111.00	100.00	99.84	97.74	94.77
FANCC	NM_000136.3	91.00	100.00	100.00	99.61	93.15
FANCD2	NM_001018115.2	110.00	100.00	100.00	99.96	99.63
FANCE	NM_021922.3	87.00	95.36	89.17	86.16	80.81
FANCF	NM_022725.4	206.00	100.00	100.00	99.86	98.80
FANCG	NM_004629.1	106.00	100.00	99.71	97.91	91.91
FANCI	NM_001113378.1	117.00	100.00	100.00	99.99	99.61
FANCL	NM_018062.3	91.00	100.00	100.00	99.95	92.89
FANCM	NM_020937.4	138.00	100.00	99.87	99.11	96.06
FERMT3	NM_178443.2	82.00	99.49	95.87	92.05	81.85
FLI1	NM_002017.5	115.00	100.00	99.89	99.53	97.78
FLT3	NM_004119.3	112.00	100.00	99.49	98.67	98.06
G6PC3	NM_138387.3	83.00	99.75	94.51	85.88	79.06
GATA1	NM_002049.4	57.00	99.23	93.53	86.02	67.31
GATA2	NM_032638.5	74.00	100.00	98.80	92.96	74.13
GFI1	NM_005263.5	69.00	100.00	99.65	98.03	88.08
GFI1B	NM_004188.7	106.00	100.00	100.00	100.00	100.00
GLRX5	NM_016417.3	33.00	81.54	65.97	53.73	38.54
GNAS	NM_080425.3	169.00	100.00	99.92	99.53	96.20
GRHL2	NM_024915.4	103.00	100.00	99.87	99.02	93.15
HAX1	NM_006118.4	109.00	100.00	100.00	99.99	96.77
HOXA11	NM_005523.6	100.00	100.00	99.41	94.74	80.77
IDH1	NM_005896.3	121.00	100.00	100.00	100.00	100.00
IDH2	NM_002168.3	119.00	98.97	94.44	91.84	91.21
IKZF1	NM_006060.6	135.00	100.00	99.82	98.86	97.74
JAK2	NM_004972.3	107.00	99.99	99.91	99.37	97.86
JAK3	NM_000215.3	91.00	99.97	99.32	97.45	91.76
KDM6A	NM_001291415.1	93.00	100.00	99.60	97.93	88.13
KIF23	NM_138555.4	109.00	100.00	99.97	99.31	94.44
KIT	NM_000222.2	113.00	100.00	100.00	100.00	99.91
KLF1	NM_006563.5	77.00	99.93	98.37	92.07	84.38
KMT2A	NM_001197104.1	159.00	99.73	99.18	98.38	96.31
KRAS	NM_004985.5	120.00	100.00	99.32	98.31	88.38
LIG4	NM_002312.3	286.00	100.00	100.00	100.00	100.00
LPIN2	NM_014646.2	106.00	100.00	99.99	99.87	99.17
LYST	NM_000081.4	130.00	100.00	99.98	99.80	98.24
MAD2L2	NM_006341.4	87.00	100.00	100.00	98.26	79.72
MECOM	NM_004991.4	158.00	100.00	99.95	99.34	95.32
MLPH	NM_024101.7	79.00	99.38	93.33	90.32	84.76
MPIG6B	NM_025260	77.00	99.28	93.06	87.40	84.12
MPL	NM_005373.3	100.00	100.00	99.99	99.91	97.99
MYD88	NM_001172567.2	102.00	100.00	100.00	99.74	92.16
MYH9	NM_002473.5	82.00	99.88	97.73	91.58	77.76
MYSM1	NM_001085487.3	131.00	99.93	98.75	96.25	93.05
NHP2	NM_017838.3	101.00	100.00	100.00	100.00	99.83
NOP10	NM_018648.3	103.00	100.00	100.00	100.00	100.00
NOTCH1	NM_017617.5	96.00	98.99	97.62	94.26	85.40

HGNC approved gene symbol	transcript	median depth	% covered >10x	% covered >20x	% covered >30x	% covered >50x
NPM1	NM_002520.6	141.00	94.61	93.42	93.23	90.43
NRAS	NM_002524.5	132.00	100.00	100.00	100.00	99.96
PALB2	NM_024675.4	239.00	100.00	100.00	99.94	99.29
PARN	NM_002582.4	101.00	100.00	100.00	99.92	97.88
PDGFRA	NM_006206.6	114.00	100.00	100.00	99.99	99.83
PHF6	NM_001015877.2	59.00	99.99	98.08	89.74	63.33
PIEZO1	NM_001142864.4	89.00	99.90	98.50	93.66	80.76
PRKACG	NM_002732.3	174.00	100.00	100.00	100.00	100.00
PTEN	NM_000314.8	152.00	100.00	100.00	100.00	99.55
PTPN11	NM_002834.4	137.00	99.19	99.17	99.17	99.17
PUS1	NM_025215.6	98.00	100.00	99.76	97.98	93.40
RAB27A	NM_183235.3	120.00	100.00	100.00	100.00	99.59
RAD21	NM_006265.3	100.00	100.00	100.00	99.92	98.46
RAD51	NM_002875.5	98.00	100.00	100.00	100.00	100.00
RAD51C	NM_058216.3	116.00	100.00	100.00	99.92	98.06
RASGRP2	NM_001098671.2	91.00	100.00	99.62	96.87	83.68
RBM8A	NM_005105.4	102.00	100.00	100.00	99.84	99.08
RGS2	NM_002923.4	91.00	100.00	100.00	99.84	98.84
RMRP	NR_003051	0.00	0.00	0.00	0.00	0.00
RPL11	NM_000975.5	103.00	100.00	100.00	100.00	96.50
RPL15	NM_001253383.3	101.00	99.89	98.96	95.97	93.10
RPL17	NM_001199343.2	149.00	100.00	100.00	100.00	99.73
RPL18	NM_000979.4	92.00	100.00	100.00	99.58	96.96
RPL26	NM_000987.5	113.00	100.00	100.00	100.00	100.00
RPL27	NM_000988.5	171.00	100.00	100.00	100.00	99.79
RPL31	NM_000993.5	151.00	100.00	100.00	100.00	100.00
RPL35A	NM_000996.4	170.00	99.88	97.95	93.28	92.58
RPL36	NM_033643.3	75.00	99.87	95.34	85.89	71.59
RPL5	NM_000969.5	115.00	99.76	97.47	94.63	91.69
RPL9	NM_001024921.3	122.00	100.00	100.00	100.00	99.56
RPS10	NM_001014.5	129.00	100.00	100.00	99.97	99.49
RPS14	NM_005617.4	136.00	100.00	100.00	100.00	100.00
RPS15	NM_001018.4	162.00	84.04	80.32	80.32	80.26
RPS15A	NM_001019.5	101.00	100.00	100.00	100.00	99.96
RPS17	NM_001021.6	0.00	0.00	0.00	0.00	0.00
RPS19	NM_001022.4	84.00	100.00	100.00	99.57	97.74
RPS24	NM_033022.4	218.00	100.00	100.00	100.00	100.00
RPS26	NM_001029.5	98.00	100.00	100.00	100.00	99.93
RPS27	NM_001030.6	110.00	100.00	100.00	99.69	97.33
RPS27A	NM_001177413.1	147.00	100.00	100.00	100.00	99.36
RPS28	NM_001031.5	33.00	99.93	89.83	59.83	26.50
RPS29	NM_001032.4	177.00	100.00	100.00	100.00	100.00
RPS7	NM_001011.4	153.00	92.30	87.48	85.87	81.52
RPS8	NM_001012.2	105.00	100.00	100.00	100.00	99.33
RTEL1	NM_001283009.2	90.00	100.00	99.89	98.36	86.89
RTL1	NM_001134888.2	180.00	100.00	99.94	99.60	98.43
RUNX1	NM_001754.4	62.00	99.92	97.26	86.93	67.08

HGNC approved gene symbol	transcript	median depth	% covered >10x	% covered >20x	% covered >30x	% covered >50x
SAMD9	NM_017654.4	305.00	100.00	100.00	100.00	100.00
SAMD9L	NM_152703.5	314.00	100.00	100.00	100.00	100.00
SBDS	NM_016038.4	131.00	100.00	100.00	100.00	100.00
SEC23B	NM_006363.6	114.00	100.00	99.88	98.62	95.62
SETBP1	NM_015559.3	149.00	99.37	98.06	96.13	92.51
SF3B1	NM_012433.3	116.00	99.89	98.67	96.62	93.73
SLC25A38	NM_017875.4	157.00	100.00	100.00	100.00	99.53
SLC37A4	NM_001164277.1	99.00	100.00	99.72	98.33	94.76
SLX4	NM_032444.4	108.00	99.95	99.40	97.80	93.27
SMC1A	NM_006306.4	90.00	99.96	99.41	98.06	92.98
SMC3	NM_005445.3	90.00	99.96	99.33	96.55	89.06
SRP72	NM_006947.4	112.00	100.00	100.00	100.00	99.89
SRSF2	NM_001195427.2	195.00	100.00	100.00	100.00	100.00
STAG2	NM_001042750.2	74.00	99.83	98.63	95.78	84.54
STEAP3	NM_182915.3	105.00	99.91	98.04	95.30	88.53
STIM1	NM_003156.3	108.00	100.00	99.98	99.61	96.79
STK4	NM_006282.5	107.00	99.61	98.36	96.88	91.64
TAZ	NM_000116.5	59.00	99.42	96.11	88.35	67.89
TERC	NR_001566	0.00	0.00	0.00	0.00	0.00
TERT	NM_198253.3	84.00	99.76	97.73	95.01	87.45
TET2	NM_001127208.2	214.00	100.00	99.97	99.60	98.45
THPO	NM_000460.4	163.00	100.00	99.87	99.02	95.94
TINF2	NM_001099274.3	157.00	100.00	100.00	100.00	99.93
TP53	NM_000546.5	130.00	100.00	100.00	100.00	100.00
TSR2	NM_058163.3	60.00	99.92	96.32	85.84	63.00
U2AF1	NM_006758.2	188.00	100.00	100.00	100.00	96.91
UBE2T	NM_014176.4	108.00	100.00	100.00	100.00	99.62
USB1	NM_024598.4	78.00	99.35	92.32	81.86	76.72
WAS	NM_000377.3	53.00	99.58	94.05	81.44	54.23
WRAP53	NM_018081.2	99.00	100.00	99.93	99.30	96.51
WT1	NM_024426.6	70.00	99.88	98.27	91.39	74.72
XRCC2	NM_005431.2	226.00	100.00	100.00	100.00	100.00
YARS2	NM_001040436.3	139.00	100.00	100.00	100.00	100.00
ZRSR2	NM_005089.3	79.00	94.19	86.42	78.72	67.92
